# Prediction Model for Diagnosis of Kawasaki Disease Using iTRAQ-Based Analysis

**DOI:** 10.3390/children8070576

**Published:** 2021-07-05

**Authors:** Ken-Pen Weng, Sung-Chou Li, Kuang-Jen Chien, Kuo-Wang Tsai, Ho-Chang Kuo, Kai-Sheng Hsieh, Shih-Hui Huang

**Affiliations:** 1 Congenital Structural Heart Disease Center, Department of Pediatrics, Kaohsiung Veterans General Hospital, Kaohsiung 813414, Taiwan; kenpenweng@yahoo.com.tw (K.-P.W.); kjchien@vghks.gov.tw (K.-J.C.); 2 School of Medicine, National Yang Ming Chiao Tung University, Taipei 11221, Taiwan; 3 Genomics and Proteomics Core Laboratory, Department of Medical Research, Kaohsiung Chang Gung Memorial Hospital, Chang Gung University College of Medicine Kaohsiung, Kaohsiung 83301, Taiwan; raymond.pinus@gmail.com; 4 Department of Research, Taipei Tzu Chi Hospital, Buddhist Tzu Chi Medical Foundation, New Taipei 23142, Taiwan; kwtsai6733@gmail.com; 5 Kawasaki Disease Center, Department of Pediatrics, Kaohsiung Chang Gung Memorial Hospital, Kaohsiung 83301, Taiwan; erickuo48@yahoo.com.tw; 6 Department of Pediatrics, Taipei Medical University-Shuang Ho Hospital, Taipei 110301, Taiwan; kshsieh@hotmail.com; 7 Department of Nursing, Fooyin University, Kaohsiung 83102, Taiwan

**Keywords:** Kawasaki disease, proteomics, prediction model

## Abstract

A quick prediction method may help confirm the diagnosis of Kawasaki disease (KD), and reduce the risk of coronary artery lesions. The purpose of this study was to evaluate potential candidate diagnostic serum proteins in KD using isobaric tagging for relative and absolute quantification (iTRAQ) gel-free proteomics. Ninety two subjects, including 68 KD patients (1.6 ± 1.2 years, M/F 36/32) and 24 fever controls with evident respiratory tract infection (2.1 ± 1.2 years, M/F 13/11) were enrolled. Medical records were reviewed for demographic and laboratory data. The iTRAQ gel-free proteomics was used to screen serum proteins completely and compare the difference between two groups followed by specific validation with ELISA. The candidate proteins and conventional laboratory items were selected for the prediction model of KD diagnosis by support vector machine. Five selected candidate proteins, including protein S100-A8, protein S100-A9, protein S100-A12, neutrophil defensin 1, and alpha-1-acid glycoprotein 1 were identified for developing the prediction model of KD diagnosis. They were used to develop an efficient KD prediction model with an area under receiver operating characteristic (auROC) value of 0.92 (95% confidence interval: 0.84, 0.98). These protein biomarkers were significantly correlated with the conventional laboratory items as follows: C-reactive protein, glutamic pyruvic transaminase, white blood count, platelet, segment and hemoglobin. These conventional laboratory items were used to develop a prediction model of KD diagnosis with an auROC value of 0.88 (95% confidence interval: 0.80, 0.96). Our result demonstrated that the prediction model with combined five selected candidate protein levels may be a good diagnostic tool of KD. Further prediction model with combined six conventional laboratory data is also an acceptable alternative method for KD diagnosis.

## 1. Introduction

Kawasaki disease (KD) is an acute systemic vasculitis, preferentially affecting Asian infants and children under 5 years of age [1,2]. Delayed detection and management of KD (especially atypical KD) may lead to a high risk of coronary artery lesions (CALs) [2,3]. The diagnosis of KD depends mainly on the clinical features. Typical diagnostic symptoms of KD include fever for at least 5 days, nonpurulent conjunctivitis, extremity edema, oral mucosal changes with red lips and strawberry tongue, cervical lymphadenopathy, and a polymorphous skin rash [2]. However, these clinical features are not objective and similar to the infectious symptoms by pathogens, resulting in difficulty in accurate diagnosis and timely treatment of KD.

Previous studies suggested the abnormal immune activation and cytokine release in the development of KD [4,5,6,7,8,9,10,11,12,13,14,15,16,17,18]. These KD related inflammatory proteins include interleukin (IL-1), tumor necrosis factor-α, IL-4, IL-6, IL-8, IL-10, IL-18, matrix metalloproteinase, tissue inhibitor of metalloproteinase and vascular endothelial growth factor which interact together during acute phase of KD [4,5,6,7,8,9,10,11,12,13,14,15,16,17,18]. However, they are not specific proteins for definite diagnosis of KD. To overcome this problem, many studies have been performed for finding useful biomarkers in the early diagnosis and management of KD [4,5,7,12,14]. However, cytokine-based models for KD diagnosis have not been developed perfectly. Rapid development of proteomics leads to its application in many disease studies, including KD [19,20,21,22]. Zhang et al. performed two-dimensional (2D) electrophoresis-based analysis to compare the serum proteomes of normal children and KD patients by identifying differentially expressed proteins before and after intravenous immunoglobulin (IVIG) therapy [19]. They identified 29 differentially expressed proteins in KD patients and found that the proteins, especially transthyretin, are potential markers for therapeutic monitoring [19]. Kimura et al. identified a total of 1879 differentially expressed proteins between acute and recovery phases of KD patients by mass spectrometry (MS)-based proteomic analysis [20]. Their results suggested leucine-rich alpha-2-glycoprotein could be used as biomarkers to facilitate KD [20]. Kuo et al. profiled the plasma antibodies by using expensive *E. coli* proteome microarray between KD and healthy subjects [21]. They found 70 proteins were shown to have high accuracy in diagnosis of KD [21]. The technique of proteomics progresses from traditional 2D-gel electrophoresis to the more efficient isobaric tagging for relative and absolute quantification (iTRAQ) method, which has emerged as a powerful tool for discovering candidate protein biomarkers in the proteomic researches [23,24]. 

A quick and efficient prediction method may facilitate the diagnosis of KD, significantly reducing the risk of CALs. The purpose of this study was to evaluate candidate diagnostic serum proteins in KD using iTRAQ gel-free proteomics, compare selected candidate proteins with conventional laboratory items, and develop the prediction model of KD diagnosis using both combined selected candidate proteins as well as conventional laboratory items.

## 2. Materials and Methods

### 2.1. Subject Enrollment

We enrolled 68 typical KD patients and 24 fever control (FC) subjects as the comparison group between 1998 and 2018 at the department of pediatrics, Kaohsiung Veterans General Hospital (KVGH), Taiwan. The inclusion criteria were as follows: (1) KD patients meeting the diagnostic criteria of KD, including fever for at least 5 days, nonpurulent conjunctivitis, extremity edema, oral mucosal changes with red lips and strawberry tongue, cervical lymphadenopathy, a polymorphous skin rash and Bacillus Calmette-Guérin (BCG) site erythema [2]. (2) FC subjects with fever (body temperature  ≥  38 °C) for at least 5 days, with evident respiratory tract infections diagnosed, and without history of KD, autoimmune disease, allergic disease or cardiovascular disease. The exclusion criteria were as follows: (1) KD patients who had no serum samples before IVIG treatment in acute stage, missing laboratory data, did not receive IVIG treatment or received initial IVIG treatment beyond 10 days of fever. (2) FC subjects who had no serum samples in acute stage, or missing laboratory data. Medical records were reviewed for age, sex, presenting symptoms, doses of IVIG treatment (2 gm/kg/dose), complications and laboratory data. Blood samples (about 1–2 mL) of KD patients were be collected before IVIG treatment in acute stage after obtaining informed consent from their guardians. The FC subjects also underwent blood sampling (about 1–2 mL) once after obtaining informed consent from their guardians. Samples were processed, separated into aliquots of 1 mL and then frozen to −80 °C until analysis of proteomics. This study was carried out after the approval of the Institutional Review Board of KVGH (IRB number: VGHKS19-CT2-22). All guardians signed the informed consent form. 

### 2.2. ITRAQ Gel-Free Proteomics

For diagnosis of KD, we first had serum samples subject to high-abundant depletion kit treatment to remove high-abundant proteins, including albumin, immunoglobulin (Ig)G, IgA, IgM, α1-acid glycoprotein, α1-antitrypsin, α2-macroglobulin, apolipoprotein A-I, apolipoprotein A-II, fibrinogen, haptoglobin and transferrin. Then, 12 serum samples from FC subjects were evenly pooled to generate two serum libraries. Twelve serum samples from KD patients in acute phase were evenly pooled to generate two serum libraries. By doing so, we collected two pooled FC and two pooled KD serum libraries. Then, the following treatment was performed using the iTRAQ reagent kit according to the manufacturer’s instructions. The samples were reduced with tris-(2-carboxyethyl) phosphine and alkylated with methyl methanethiosulfonate. Trypsin was added to the samples, and the solutions were incubated at 37 °C overnight. The peptide mixtures were then labeled with the iTRAQ 4-plex reagent. Two FC sample libraries were labeled with iTRAQ 114 and 115. Two KD sample libraries were labeled with iTRAQ 116 and 117.

To examine the difference of serum proteins between the KD and FC groups by iTRAQ, samples were be pooled for analysis in each group. Serum proteins with 1.5 fold change were selected as candidate biomarkers for KD diagnosis. 

### 2.3. ELISA Validation for Selected Candidate Proteins

Among the proteins detected by iTRAQ gel-free proteomics, we selected five most fold-change candidate proteins as follows: protein S100-A8 (S100A8), protein S100-A9 (S100A9), protein S100-A12 (S100A12), neutrophil defensin 1 (DEFA1) and alpha-1-acid glycoprotein 1 (ORM1) for further ELISA validation in both KD and FC groups. The procedure for ELISA was performed according to the manufacturer’s instructions.

### 2.4. Support Vector Machine (SVM) Alignment for Prediction Model of KD Diagnosis

SVM is one type of machine learning algorithm specifically good in dealing with binary classification problem, e.g., disease versus health, control versus treatment and positive versus negative [25]. When applying SVM in the binary classification jobs, the users must first prepare the positive and negative sets and the individuals in sets must have numerical variables (also called vectors). For example, we collected positive (KD) and negative (FC) sets in this study. Every subject had numerical values of five selected candidate protein levels and the following conventional laboratory data including c-reactive protein (CRP), glutamic oxaloacetic transaminase (GOT) and glutamic pyruvic transaminase (GPT), etc. Then, by inputting the data of positive and negative sets into SVM, SVM generated a classification model. With this classification model, an unknown case (waiting for being classified into positive or negative set) can be classified. A machine learning algorithm usually reports an area under the receiver operating characteristic curve (auROC) value to represent the overall performance [26]. The X- and *Y*-axis of an auROC plot denote false positive rate and true positive rate, respectively.

### 2.5. Statistical Analysis

Continuous variables are expressed as means with standard deviation. Categorical variables are presented as absolute numbers and percentages. Comparison of continuous variables (or categorical variables) between two groups was carried out using the two-tailed unpaired t-test (or chi-square or Fisher’s test as appropriate). Correlation analysis was carried out using the Pearson correlation method. SVM was used for prediction model of KD diagnosis. An auROC value with 95% confidence interval (CI) was calculated. A *p*-value < 0.05 was considered statistically significant.

## 3. Results

In this study, we enrolled 68 KD patients (1.8 ± 1.2 years, M/F 36/32) and 24 FC subjects (2.3 ± 1.3 years, M/F 10/24). There was no significant difference between two groups in terms of age, sex and blood sampling time since fever onset. There was a significant difference in the presence of signs between two groups in terms of nonpurulent conjunctivitis, fissured lips or strawberry tongues, polymorphous rash, extremity edema or erythema and BCG site erythema except fever ≥ 5 days as well as cervical adenopathy (Table 1).

Table 2 showed the conventional laboratory data in two groups. Compared to the FC subjects, KD patients had significantly higher CRP (*p* < 0.001), GPT (*p* < 0.001), white blood count (WBC) (*p* < 0.001), platelet (*p* < 0.001) and segment (*p* < 0.01), but lower hemoglobin (Hgb) (*p* < 0.001). There was no significance between groups in terms of GOT level (*p* > 0.05).

Table 3 showed S100A8, S100A9, S100A12, DEFA1 and ORM1 levels in two groups. Compared to the FC subjects, KD patients had significantly higher S100A8 (717.9 ± 154.3 vs. 413.2 ± 223.9 ng/mL, *p* < 0.001), S100A9 (119.8 ± 32.9 vs. 70.6 ± 43.8 ng/mL, *p* < 0.001), S100A12 (798.4 ± 603.9 vs. 337.5 ± 410.8 ng/mL, *p* < 0.001), ORM1 (5375.1 ± 1490.6 vs. 3047.7 ± 1137.6 ng/mL, *p* < 0.001) and DEFA1 (2611.7 ± 2535.2 vs. 1193.9 ± 1344.4 μg/mL, *p* < 0.001).

Table 4 showed the correlation between the conventional laboratory items and 5 selected candidate proteins. S100A8 was significantly correlated with CRP (*p* < 0.001), WBC (*p* < 0.0001), platelet (*p* < 0.0001) and segment (*p* < 0.0001) except GOT, GPT, and Hgb. S100A9 was significantly correlated with CRP (*p* < 0.01), GPT (*p* < 0.05), WBC (*p* < 0.0001), platelet (*p* < 0.0001) and segment (*p* < 0.0001) except GOT and Hgb. S100A12 was significantly correlated with GOT (*p* < 0.05), WBC (*p* < 0.001), platelet (*p* < 0.01), segment (*p* < 0.001) and Hgb (*p* < 0.05) except CRP and GPT. DEFA1 was significantly correlated with CRP (*p* < 0.05), GPT (*p* < 0.05), segment (*p* < 0.01) and Hgb (*p* < 0.01) except GOT, WBC, and platelet. ORM1 was significantly correlated with CRP (*p* < 0.0001), WBC (*p* < 0.05), platelet (*p* < 0.05), segment (*p* < 0.05) and Hgb (*p* < 0.01) except GOT and GPT.

**Figure 1:**
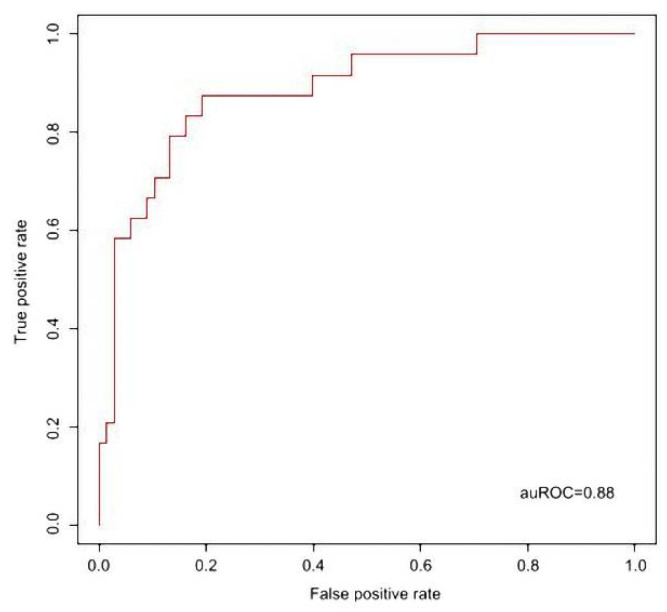
The performance of KD prediction model developed with the conventional laboratory items. Using the values of six conventional laboratory items, we developed a KD prediction model with auROC = 0.88 (95% CI: 0.80, 0.96).

**Figure 2:**
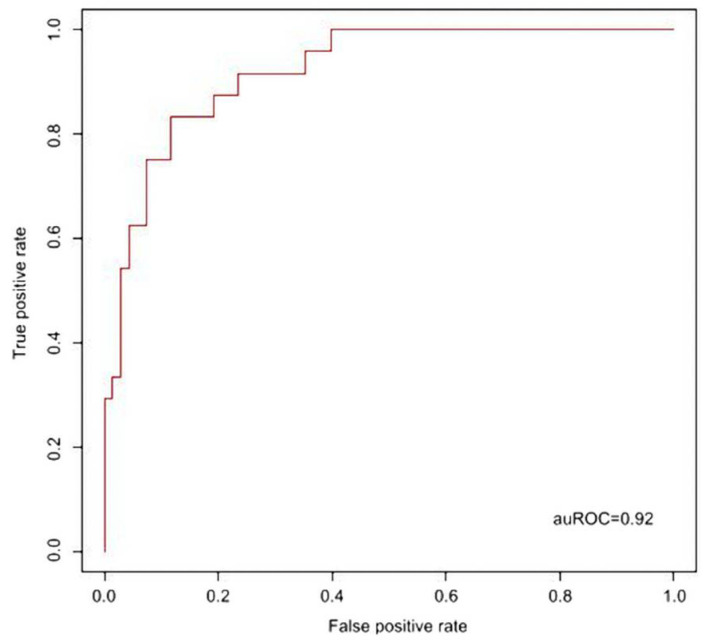
The performance of KD prediction model developed with 5 selected candidate proteins. Using the values of five selected proteins, we developed a KD prediction model with auROC = 0.92 (95% CI: 0.84, 0.98).

We inputted the values of the six conventional laboratory items (CRP, GPT, WBC, Hgb, platelet and segment) without GOT (no significant difference between two groups) into SVM and conducted a 10-fold cross validation to derive the optimal parameters, namely gamma = 0.0039 and cost = 8. Then, with the specified gamma and cost parameters, we used the values of the conventional laboratory items to develop a KD prediction model. As shown in Figure 1, this prediction model had an auROC value 0.88 (95% CI: 0.80, 0.96). In addition, we also conducted similar analysis based on the profiles of five candidate proteins (S100A8, S100A9, S100A12, DEFA1 and ORM1). As a result, this protein concentration-based prediction model had an auROC value 0.92 (95% CI: 0.84, 0.98) (Figure 2, gamma = 0.0625 and cost = 1). The auROC values of the two models overlapped so that both models are useful for early diagnosis of KD.

## 4. Discussion

Our study demonstrated that the prediction model with combined S100A8, S100A9, S100A12, DEFA1 and ORM1 levels can help confirm the KD diagnosis with an auROC value 0.92. The combined six conventional laboratory data (CRP, GOT, GPT, WBC, platelet, segment and Hgb) can help confirm the KD diagnosis with an auROC value 0.88. The auROC values of the two models overlapped so that both models are useful for early diagnosis of KD. In the clinical practice, the prediction model with combined conventional laboratory data is a more easy and cost-effective way for KD diagnosis than that with combined selected candidate protein levels. Few cytokine based platforms for KD diagnosis have been reported and underline the potential value of our prediction model using combined six conventional laboratory data or five selected candidate protein levels [27,28]. In this series, we further documented the significant association of the five selected candidate proteins and conventional laboratory items.

The previous dada on the KD diagnosis based on proteomics are limited and inconsistent. Zhang et al. reported that 29 differentially expressed proteins in KD patients and found that the proteins, especially Transthyretin, are potential markers for therapeutic monitoring [19]. Kimura et al.’ results suggested leucine-rich alpha-2-glycoprotein could be used as biomarkers to facilitate KD [20]. Kuo et al. found 70 proteins were shown to have high accuracy in diagnosis of KD [21]. Compared to these previous studies using traditional proteomics [19,20,21], our study used iTRAQ method and showed it as a powerful tool for discovering candidate proteins in KD diagnosis. Further multi-center studies are needed to evaluate the feasibility of iTRAQ method in selecting diagnostic or therapeutic targets in KD.

The role of five selected candidate proteins for KD diagnosis in this series had been reported previously. Fu et al. reported that S100A12 expression on the circulating endothelial cell (CEC) surface increased significantly in patients with KD [29]. Armaroli et al. suggested S100A12 appears to activate human coronary artery endothelial cells in an IL-1β-dependent manner [30]. Li et al.’ study showed S100A12 promoted both freshly clinically isolated neutrophils and neutrophil-like cells to infiltrate through the endothelial layer in vitro [28]. Further in vitro study by Li et al. implied that S100A12 could be a potential therapeutic target for KD [28]. Lech et al. reported elevated levels of plasma calprotectin months to decades after acute KD and infiltration of cells expressing S100A8 and S100A9 in vascular tissues suggest ongoing, subclinical inflammation [31]. DEFA1 and ORM1 were first reported to be significantly correlated with Kawasaki disease in acute stage according to Li et al.’ study [28]. ORM1 is very significantly correlated to CRP in this series. Both ORM1 and DEFA1 are related to inflammation [32,33] and therefore speculated to play a role in the KD pathogenesis. Further studies are required to evaluate the role of these proteins, especially DEFA1 and ORM1, in the pathogenesis of KD.

There are some limitations of this study. First, the big limitation is that the comparison group cannot completely meet the major signs of KD except fever duration ≥5 days and cervical adenopathy. We matched two groups in terms of age, sex and blood sampling time since fever onset for decreasing analytic deviation. Second, the comparison group comprised FC subjects with heterogeneous respiratory tract infections. The extrapolation and validation of our results are therefore limited. Third, this is a single-center investigation with limited number of patients. A multi-center study with a large cohort is suggested.

## 5. Conclusions

In conclusion, our result demonstrated that the prediction model with combined S100A8, S100A9, S100A12, DEFA1 and ORM1 levels may be a good diagnostic tool of KD. Further prediction model with combined six conventional laboratory data (CRP, GPT, WBC, Hgb, platelet and segment) is an acceptable alternative method for KD diagnosis.

## Figures and Tables

**Table 1:** The demographic data of subjects.

	KD (*n* = 68)	FC (*n* = 24)	*p*
Age (years)	1.8 ± 1.2	2.3 ± 1.3	NS
M/F	36/32	10/24	NS
Blood sampling time since fever onset (day)	5.8 ± 0.8	5.7 ± 0.9	NS
Fever duration ≥ 5 days (%)	100	100	NS
Nonpurulent conjunctivitis (%)	96	42	<0.001
Fissured lips or strawberry tongues (%)	91	37	<0.001
Polymorphous rash (%)	88	33	<0.001
Cervical adenopathy (%)	53	50	NS
Extremity edema or erythema (%)	79	12	<0.001
BCG site erythema (%)	63	0	<0.001

KD = Kawasaki disease; FC = fever control; BCG = Bacillus Calmette-Guérin; NS = nonsignificant.

**Table 2:** Conventional laboratory data in the KD patients and FC subjects.

	KD (*n* = 68)	FC (*n* = 24)	*p*
CRP (mg/dL)	6.8 ± 5.4	2.9 ± 3.9	<0.001
GOT (U/L)	66.7 ± 118.1	51.5 ± 14.5	NS
GPT (U/L)	81.7 ± 87.2	25.9 ± 11.9	<0.001
WBC (/mm^3^)	11897.4 ± 4195.7	7952.8 ± 3394.2	<0.001
Hgb (g/dL)	11.1 ± 1.1	11.9 ± 1.1	<0.01
platelet (×1000/mm^3^)	352.5 ± 122.3	240.8 ± 100.6	<0.001
Segment (%)	52.9 ± 17.0	39.3 ± 21.2	<0.01

KD = Kawasaki disease; FC = fever control; CRP = C-reactive protein; GOT = glutamic oxaloacetic transaminase; GPT = glutamic pyruvic transaminase; WBC = white blood count; Hgb = hemoglobin, NS = nonsignificant.

**Table 3:** Five selected candidate proteins in the KD patients and FC subjects.

	KD (*n* = 68)	FC (*n* = 24)	*p*
S100A8 (ng/mL)	717.9 ± 154.3	413.2 ± 223.9	<0.001
S100A9 (ng/mL)	119.8 ± 32.9	70.6 ± 43.8	<0.001
S100A12 (ng/mL)	798.4 ± 603.9	337.5 ± 410.8	<0.001
ORM1 (ng/mL)	5375.1 ± 1490.6	3047.7 ± 1137.6	<0.001
DEFA1 (μg/mL)	2611.7 ± 2535.2	1193.9 ± 1344.4	<0.001

KD = Kawasaki disease; FC = fever control; DEFA1 = neutrophil defensin 1; ORM1 = alpha-1-acid glycoprotein 1.

**Table 4:** Correlation of the conventional laboratory items and five selected candidate proteins.

	CRP	GOT	GPT	WBC	Platelet	Segment	Hgb
S100A8	0.37 ***	0.07	0.19	0.42 ****	0.41 ****	0.53 ****	−0.19
S100A9	0.33 **	0.12	0.21 *	0.44 ****	0.41 ****	0.55 ****	−0.02
S100A12	0.19	0.24 *	0.13	0.37 ***	0.33 **	0.36 ***	−0.21 *
DEFA1	0.26 *	0.19	0.24 *	0.15	0.18	0.26 **	−0.27 **
ORM1	0.47 ****	0.06	0.19	0.24 *	0.25 *	0.37 ***	−0.33 **

CRP = C-reactive protein; GOT = glutamic oxaloacetic transaminase; GPT = glutamic pyruvic transaminase; WBC = white blood count; Hgb = hemoglobin; DEFA1 = neutrophil defensin 1; ORM1 = alpha-1-acid glycoprotein 1. * *p* < 0.05, ** *p* < 0.01, *** *p* < 0.001, **** *p* < 0.0001.
